# Radiomic Applications on Digital Breast Tomosynthesis of BI-RADS Category 4 Calcifications Sent for Vacuum-Assisted Breast Biopsy

**DOI:** 10.3390/diagnostics12040771

**Published:** 2022-03-22

**Authors:** Benedetta Favati, Rita Borgheresi, Marco Giannelli, Carolina Marini, Vanina Vani, Daniela Marfisi, Stefania Linsalata, Monica Moretti, Dionisia Mazzotta, Emanuele Neri

**Affiliations:** 1 Department of Translational Research, University of Pisa, 56126 Pisa, Italy; bene.favati@gmail.com (B.F.); emanuele.neri@med.unipi.it (E.N.); 2 Unit of Medical Physics, Azienda Ospedaliero-Universitaria Pisana, Via Roma 67, 56126 Pisa, Italy; rita.borghe@gmail.com (R.B.); m.giannelli@ao-pisa.toscana.it (M.G.); marfisi.daniela@gmail.com (D.M.); stefania.linsalata@ao-pisa.toscana.it (S.L.); 3 S.D. Radiologia Senologica, Azienda Ospedaliero-Universitaria Pisana, Via Roma 67, 56125 Pisa, Italy; c.marini@ao-pisa.toscana.it (C.M.); mo.moretti@ao-pisa.toscana.it (M.M.); d.mazzotta@ao-pisa.toscana.it (D.M.); 4 Italian Society of Medical and Interventional Radiology (SIRM), SIRM Foundation, Via della Signora 2, 20122 Milan, Italy

**Keywords:** breast calcifications, digital breast tomosynthesis, radiomics, diagnosis

## Abstract

Background: A fair amount of microcalcifications sent for biopsy are false positives. The study investigates whether quantitative radiomic features extracted from digital breast tomosynthesis (DBT) can be an additional and useful tool to discriminate between benign and malignant BI-RADS category 4 microcalcification. Methods: This retrospective study included 252 female patients with BI-RADS category 4 microcalcifications. The patients were divided into two groups according to micro-histopathology: 126 patients with benign lesions and 126 patients with certain or possible malignancies. A total of 91 radiomic features were extracted for each patient, and the 12 most representative features were selected by using the agglomerative hierarchical clustering method. The binary classification task of the two groups was carried out by using four different machine-learning algorithms (i.e., linear support vector machine (SVM), radial basis function (RBF) SVM, logistic regression (LR), and random forest (RF)). Accuracy, sensitivity, sensibility, and the area under the curve (AUC) were calculated for each of them. Results: The best performance was achieved using the RF classifier (AUC = 0.59, 95% confidence interval 0.57–0.60; sensitivity = 0.56, 95% CI 0.54–0.58; specificity = 0.61, 95% CI 0.59–0.63; accuracy = 0.58, 95% CI 0.57–0.59). Conclusions: DBT-based radiomic analysis seems to have only limited potential in discriminating benign from malignant microcalcifications.

## 1. Introduction

Breast cancer is the most diagnosed female tumor. According to recent estimates from the International Agency for Research on Cancer (IARC), about 2.3 million women were diagnosed with breast cancer in 2020, exceeding the incidence of lung cancer for the first time. Furthermore, breast cancer is the leading cause of death for female tumors and the fifth cause of death for cancer in the general population [1,2,3]. These data suggest that early diagnosis and immediate treatment are needed to reduce morbidity and mortality and increase survival in these patients. Mammography is the gold standard screening technique for the diagnosis of breast cancer, which, in fact, allows an early diagnosis of non-palpable neoplasms, some of which are discovered in an early stage as carcinoma in situ [1,2,3,4,5]. About 55% of non-palpable tumors are detected thanks to the presence of microcalcifications; those, existing in about 30% of malignant breast tumors, represent the main form of presentation of the ductal carcinoma in situ (DCIS), allowing the diagnosis of DCIS in 85–95% of the cases [6]. The microcalcifications are the product of altered cell metabolism, related either to malignancy as breast cancer or to benign pathology as inflammation or an infection of the mammary gland. The Breast Imaging Reporting and Data System (BI-RADS) of the American College of Radiology has standardized the qualitative description and the management of the imaging findings. Regarding the microcalcifications, the BI-RADS V edition suggests the use of morphology and distribution descriptors to insert the findings in an assessment category [7]. In particular, amorphous microcalcifications in a grouped, linear, or segmental distribution, a single group of coarse heterogeneous microcalcifications, and those with fine pleomorphic and fine linear or fine-linear branching morphology are considered as BI-RADS assessment category 4. BIRADS 4 includes atypical findings that are suspicious enough for malignancy to justify a recommendation to biopsy. A finding included in this category has a 2–95% chance of being a neoplasia. Regarding the detection of the microcalcifications, mammography is a test with high sensitivity (about 95%) but low specificity (about 41%), with a positive predictive value (PPV) of less than 30% [8,9]. In fact, most of the microcalcifications biopsied (70–80%) are histologically benign [10,11]. Therefore, most of the biopsies executed for suspicious microcalcifications could be avoided. Based on the above considerations, researching a diagnostic tool capable of reducing the percentage of false positives is important. This hypothetical tool should overcome the large use of biopsy, which is an invasive, not risk-free, and expensive technique. Radiomics is a new research tool well suited to this scenario. Indeed, radiomics represents an advanced analytic methodology that extracts quantitative features from biomedical images to generate imaging biomarkers. According to the literature, radiomics could be applied to mammographic images in many ways. For example, Tagliafico et al. and Sakay et al. have proposed new methods for the automatic classification of benign and malignant lesions on digital breast tomosynthesis [12,13]; Son et al. and Ma et al. have tried to predict the breast cancer molecular subtypes using quantitative radiomic features extracted from mammography [14,15]; Zhou et al. have evaluated the state of HER-2 in patients with breast cancer using radiomic features from mammography [16]; Lei et al. have proposed a radiomic model based on mammographic images and clinical risk factors to predict the malignant or benign nature of BI-RADS category 4 microcalcifications [17]; Chen et al. have assessed the capability of a multimodal radiomic approach, based on the integration of mammographic and contrast-enhanced magnetic resonance images, to identify non-palpable malignant lesions presented as BI-RADS category 3–5 microcalcifications [18]. Furthermore, dedicated breast CT has been evaluated as a non-invasive and supportive tool in the diagnosis of breast cancer [19]. Our study aims to evaluate whether the radiomic analysis of digital breast tomosynthesis can foretell the histopathological report and, consequently, the management of BI-RADS category 4 microcalcifications addressed to stereotactic biopsy: benign microcalcification intended for follow up or certain/possible malignant microcalcifications intended for surgical excision.

## 2. Materials and Methods

### 2.1. Study Design and Population

This retrospective study was performed in the framework of the HORIZON 2020 projects CHAIMELEON (Accelerating the lab to the market transition of AI tools for cancer management) and EuCanImage (Towards a European cancer imaging platform for enhanced artificial intelligence in oncology) [20,21]. In both projects, multiple tasks are dedicated to the extraction and analysis of radiomic features in breast cancer. Two-hundred-eighty female patients (age range 24–85 years; mean 55.28) sent for vacuum-assisted breast biopsy (VABB) for BI-RADS category 4 microcalcifications were evaluated in our institution from November 2018 to August 2020.

The inclusion criteria were:

- Detection of microcalcification at the most recent mammography.
- Microcalcifications classified by a radiologist as BI-RADS category 4.
- Execution of VABB in high-definition breast tomosynthesis.
- Histological examination of the specimens in our institution.

The exclusion criteria were:

- Presence of a mass or an architectural distortion associated with microcalcification.
- Allergic reaction to the local anesthetic.
- Hemorrhagic diathesis or the impossibility of discontinuing an antiplatelet/anticoagulant therapy.
- Technical unfeasibility of the execution of VABB (not cooperative patients, thin breast, microcalcifications too close to the chest wall or near the skin or in the breast tissue of the axillary tails).

Therefore, 28 patients were excluded (7 patients had a mass associated with the microcalcifications; 11 patients had hemorrhagic diathesis; 4 patients could not withdraw the anticoagulant therapy; 6 patients had technical barriers for the execution of the VABB), yielding a total of 252 patients enrolled in the study. Written informed consent to the VABB for the diagnostic workup of breast microcalcifications had been obtained from all patients, and institutional review board approval was waived due to the retrospective nature of the study. The histopathological examination was classified into five categories according to the European guidelines [22]:

- B1 (Uninterpretable/Normal tissue only): indicates a core of normal tissue or an uninterpretable specimen, for example, due to an excessive crush artifact or composed of blood clots only.
- B2 (Benign lesion): indicates the presence of a benign abnormality such as fibroadenomas, fibrocystic changes, sclerosing adenosis, and duct ectasia.
- B3 (Lesion of uncertain malignant potential): indicates lesions that may provide benign histology in needle core biopsy (NCB) but are either known to show heterogeneity or to have an increased risk of associated malignancy (e.g., papillary lesions, radial scar/complex sclerosing lesion, lobular intraepithelial neoplasia, atypical epithelial proliferation of ductal type and phyllodes tumor).
- B4 (Suspicious for malignancy): indicates apparently neoplastic cells contained within blood clots or adherent to the outer aspect of the sample or technical problems such as crushed or poorly fixed cores which contain probable carcinoma.
- B5 (Malignant): indicates unequivocal malignancy on NCB. Further categorization into in situ (B5a) and invasive malignancy (B5b) should be undertaken whenever possible.

According to the histopathologic report of the digital breast tomosynthesis-guided vacuum-assisted breast biopsy (DBT-VABB) samples, the patient cohort was divided into two groups (Table 1):

- Benign group: 126 patients with category B2 addressed to imaging follow-up.
- Malignant group: 126 patients with category B3 (45 patients), B5a (63 patients), and B5b (18 patients) addressed to surgical excision.

None of the patients had a micro-histological diagnosis of category B1 or B4. There were no cases of clinical and/or radiological suspicion that required a repetition of the biopsy of category B2, according to the European and Italian guidelines [22,23]. All the 45 patients categorized as B3 received a surgical excision after a discussion at a preoperative multidisciplinary meeting, according to the international and national recommendations [22,23,24,25].

### 2.2. Imaging Protocol

All the biopsies were performed after informing the patient about the procedure, after verifying the absence of contraindications such as allergic reactions and drug therapies, and after receiving the written informed consensus. The VABB was conducted using the Hologic Selenia Dimensions System^®^ with the Affirm™ breast biopsy guidance system and an ATEC 9 gauge needle. The target lesion was localized under stereotactic guidance. An open system was used, and 12 frustules of tissue were sampled, rotating the single needle up to 360-degrees along the needle axis. Each procedure was followed by the insertion of a nonmagnetic radiopaque marker to localize the biopsied region. A post VABB radiogram was always performed to report the correct execution of the biopsy, verifying the correct position of the marker and the possible presence of residual microcalcifications. Radiograms with a magnification of the samples were performed to verify the presence of the microcalcifications within the specimens; the frustules were fixed on 10% buffered formalin and were divided into those with microcalcification and those without and, finally, were sent to the pathologist.

### 2.3. Image Segmentation and Feature Extraction

**Figure 1:**
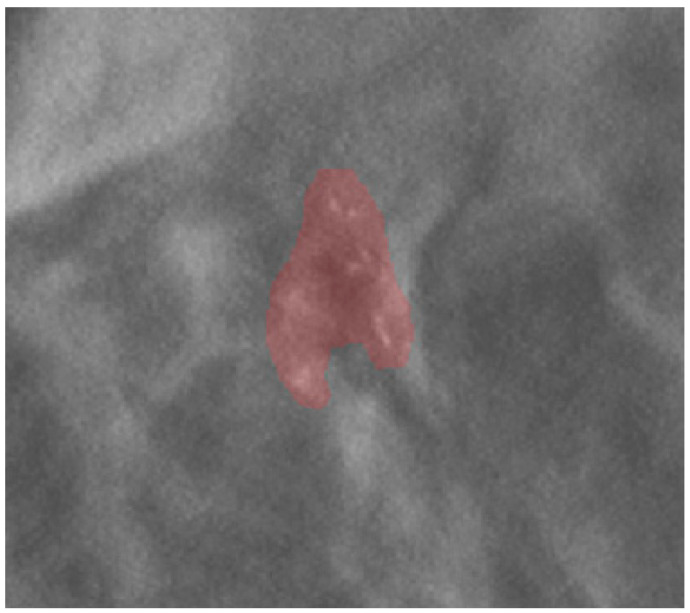
An example of ROI segmentation on a centering tomosynthesis image acquired in the biopsy session.

Images and the patient’s data were anonymized before archival in the institutional research repository. The mammographic images were examined by a radiologist with 20 years of experience in breast imaging. The radiologist traced the region of interest (ROI) by manual segmentation, using the open-source software ITK-SNAP (version 3.6.0, www.itksnap.org, last access 14 May 2021), on the centering tomosynthesis of the calcifications performed in the VABB session. The ROI included the area related to the microcalcifications (Figure 1). The radiologist was blinded to the histopathological report.

Radiomic features were calculated using PyRadiomics v3.0.1 [26], an open-source python package for the extraction of radiomics features from medical imaging in compliance with the Image Biomarker Standardization Initiative (IBSI) [27]. A total of 91 features were extracted from each ROI: 18 first-order features (first-order class), 22 gray level co-occurrence matrix features (GLCM class), 14 gray level dependence matrix features (GLDM class), 16 gray level run length matrix features (GLRLM class), 16 gray level size zone features (GLSZM class), and 5 neighboring gray-tone difference matrix features (NGTDM class). Given that radiomic features were estimated from an area indicative of the calcification region and not from each single microcalcification, shape features were not included in this work. Texture features (i.e., radiomic features belonging to the GLCM, GLDM, GLRLM, GLSZM, and NGTDM classes) were computed according to the Chebyshev norm with a distance of 1 pixel. GLCM and GLRLM features were estimated from each 2D directional matrix (i.e., at 0°, 45°, 90°, and 135°) and then averaged over 2D directions and slices. Prior to radiomic features estimation, a quantization of the image intensities inside the ROI was carried out using a fixed number of 80 bins [28,29,30]. This intensity discretization method was used according to IBSI recommendation in order to obtain a normalizing effect inside the ROI. No voxel interpolation was performed.

### 2.4. Feature Selection

Feature selection and classification were implemented in Python environment (version 3.8.5) with the scikit-learn library (version 24.2) [31]. To avoid overfitting, an unsupervised feature selection was performed to identify a subset of nonredundant radiomic features. The number of the selected radiomic features was chosen so that the ratio between the number of data observations (i.e., subjects presenting malignant lesions) to features was 10:1 [32,33]. Accordingly, an agglomerative hierarchical clustering with average group linkage and Pearson’s correlation as the dissimilarity measure was implemented to identify 12 clusters. In each cluster, the radiomic feature with the minimum average distance (in terms of Pearson’s correlation), relative to the other features of the same cluster, was selected as the representative feature, leading to 12 representative features out of the 91 extracted radiomic features.

### 2.5. Classification

The binary classification task (i.e., benign or malignant breast lesion, based on histopathology) was carried out by employing four supervised machine-learning (ML) algorithms: linear support vector machine (SVM), radial basis function (RBF) SVM, logistic regression (LR), and random forest (RF). It is worth noting that the RF model was trained with all the 91 radiomic features, while the other classification models (i.e., SVM, RBF-SVM, and LR) were trained exploiting only the 12 representative radiomic features. Indeed, RF classifiers automatically compute the relevance score of each feature in the training phase and select a subset of features at each tree node. Training and validation were performed according to the nested k-fold cross-validation (CV) method (with 5 folds in the outer CV loop and 3 folds in the inner CV loop). Hyperparameters were optimized using a grid search in the inner 3-fold CV loop, while the outer CV loop was used to estimate the performances of the model fitting procedure. Specifically, for each classification algorithm, the mean accuracy, mean sensitivity, and mean sensibility were measured across the different folds of the outer CV loop. These values were then averaged over 100 repetitions of the entire nested k-fold CV, giving a single mean accuracy value and a single mean AUC value per algorithm. The use of nested k-fold CV allows us to perform model training independently from the hyperparameters optimization; therefore, it prevents overfitting or incorrect estimates of generalization.

## 3. Results

### 3.1. Features Selection

**Figure 2:**
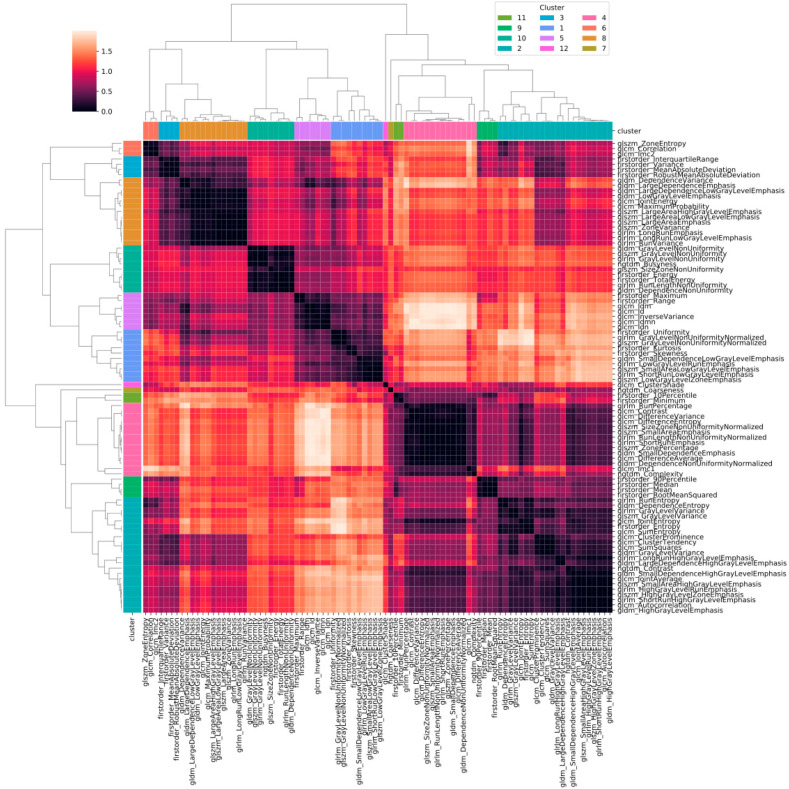
Heatmap of the correlation between pairs of radiomic features. Values in the heatmap correspond to (1 − r), where r is Pearson’s correlation coefficient.

**Figure 3:**
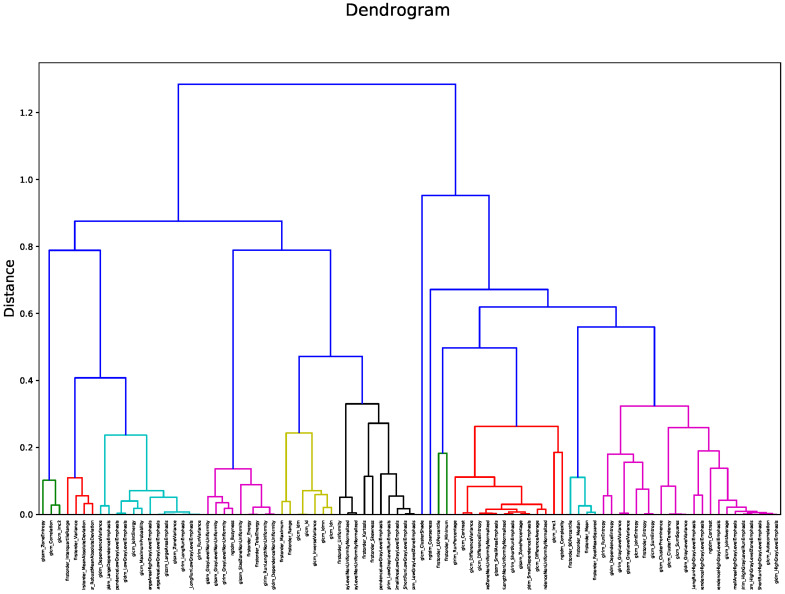
Dendrogram output for hierarchical clustering. The 12 identified clusters are highlighted in different colors.

Figure 2 shows the heatmap in which the correlation between pair of radiomic features is highlighted. The order of the radiomic features inside the heatmap reflects the output of the hierarchical clustering, with features belonging to the same cluster displayed close to each other. Figure 3 shows in greater detail which of the 12 clusters each radiomic feature belongs to. The 12 radiomic features identified as the most representative from each cluster were: MeanAbsoluteDeviation, RootMeanSquared, and Minimum from the first order class; InverseDifferenceNormalised (Idn), Correlation, and ClusterShade from the GLCM class; LowGrayLevelRunEmphasis and LongRunEmphasis from the GLRLM class; HighGrayLevelZoneEmphasis and GrayLevelNonUniformity from the GLSZM class; SmallDependenceEmphasis from the GLDM class; Strength from the NGTDM class.

### 3.2. Classification

Performances obtained from the four classification methods according to a nested 5-fold cross-validation have been summarized in Table 2. This table shows the mean and the 95% confidence interval (CI) of the accuracy, sensitivity, specificity, and AUC obtained with each classifier.

**Figure 4:**
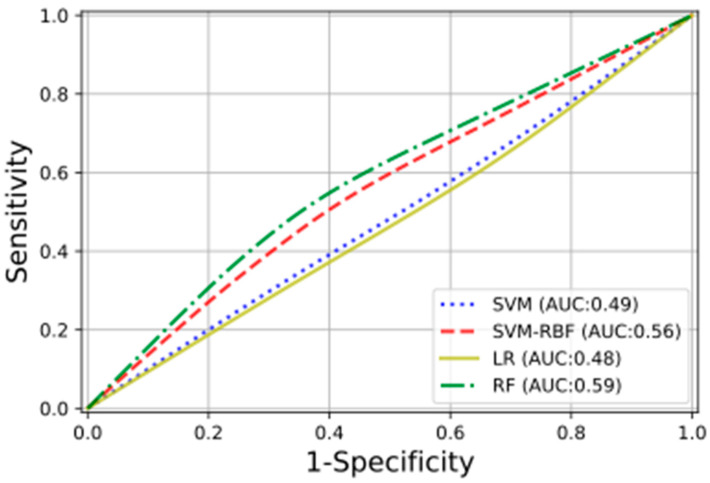
The plot presents the ROC curves, with the relative AUCs obtained using the following classifiers: linear support vector machine (SVM), radial basis function support vector machine (SVM-RBF), logistic regression (LR), and random forest (RF).

In Figure 4 are shown, with different colors and lines, the receiver operating characteristic (ROC) curves obtained for the studied ML classifiers. For three of the four classification methods, the training was performed on the selected radiomic features; for the fourth, the RF ones, all the calculated radiomic features were used. The RF classifier obtained the highest testing performance, with an AUC of 0.59.

## 4. Discussion

To date, the definitive diagnosis of suspicious breast lesions detected in imaging necessarily requires a histopathologic examination and, therefore, a biopsy sample. Considering that the majority of the suspicious microcalcifications addressed to biopsy (70–80%) turn out to be benign at the histopathological examination, many of these biopsies could be avoided. As a matter of fact, VABB is the gold standard for the sampling of microcalcifications, but it presents some disadvantages: it is invasive and unpleasant for the patients; carries risks and complications such as pain, bleeding, and infections; is expensive; and requires specialized equipment and a dedicated team [34]. Radiomics could be evaluated as a non-invasive supportive tool for a better assessment of calcifications that deserve a VABB for the purpose of reducing the rate of avoidable biopsies.

Lindfors et al. demonstrated that dedicated breast CT could be a non-invasive and comfortable instrument that supports the detection of breast tumor; however, it is less available than radiomics and still has some limitations in evaluating microcalcifications morphology compared to mammography [19]. Therefore, we focused on the radiomics applied to mammography. Accordingly, this study investigated whether quantitative radiomic features extracted from DBT could be an additional and useful tool to discriminate between benign and malignant BI-RADS category 4 microcalcification and, consequently, reduce the number of false-positive findings. Radiomic features were extracted from the centering tomosynthesis performed in the VABB session. The results of our study show that after applying ML classifiers to select the most representative features to differentiate benign from malignant microcalcifications, the highest testing performance had an AUC of only 0.59; therefore, it seems possible that radiomic features extracted from DBT do not strongly discriminate between benign and malignant microcalcifications. These results could be related to the fact that the region of interest included the whole area of microcalcifications and not every single calcification; this means the exclusion of the shape features. In this regard, Chen S. et al. have evaluated whether a multimodal radiomic model could identify malignant non-palpable breast lesions presenting as BI-RADS category 3–5 microcalcifications [18]. Unlike us, they segmented every single calcification and, therefore, considered shape and distribution as predictive features, obtaining a greater AUC (0.834). In our study, we assumed that the entire area that includes all the calcifications is more representative of the neoplasm, with respect to the area that includes a single calcification. In this regard, Holland et al. reported that the size of a DCIS, especially the low-grade type, evaluated by measuring the extent of microcalcifications using mammography, frequently underestimates the histological tumor size [35]. Berger et al., considering the microcalcifications, have shown that the extension of DCIS in histology specimens, compared to DBT, was 17.9% larger [36]. In fact, the Pathology Reporting of Breast Disease, if the excision has been undertaken for calcification, suggests we include in the histopathological evaluation the main area of calcification and the adjacent tissue to avoid the underestimation of the size of a lesion [37]. Thus, we decided to segment not every single calcification but the entire area in which they are included. Furthermore, Chen S. et al. demonstrated a better predictive value of the radiomic model when the mammographic data were integrated with those of dynamic contrast-enhanced magnetic resonance imaging (DCE-MRI) data; in this regard, a multimodality approach is likely to improve the diagnostic efficiency of the radiomics model; however, we chose to not consider data from DCE-MRI since it is a modality that is difficult to apply in the clinical evaluation of all patients with suspicious microcalcifications because of its high costs, limited availability, and risks related to the contrast agent. Therefore, in our opinion and considering the aim of our study, it is not worth applying radiomics on DCE-MRI to study microcalcifications. Lei C. et al. have tried to construct and validate a radiomic model that uses mammography-based imaging data combined with clinical data to discriminate benign from malignant BI-RADS category 4 microcalcifications [17]. While they too used an ROI that included a full-calcification related area, they extracted data from 2 dimensional (2D) mammographic images, obtaining an AUC of 0.80. In contrast, in our work, we extracted data from tomosynthesis, which provides a series of images (thin slices) that have less overlap of the structures within the breast compared to the 2D mammography. Hence, the ROI in DBT might be more representative of the lesion compared to 2D mammography. Lei et al. also demonstrated that the integration of images data with the menopausal state did not significantly improve the prediction value of the classification model. Therefore, the fact that we did not integrate imaging data with the menopausal state has probably not appreciably influenced our results. Nevertheless, other clinical risk factors could be integrated with radiomic data to better evaluate their influence on the classification models. First, a family history of breast tumor, which has a major impact on the probability of having breast cancer. Second, the use of menopausal hormone therapy, in particular combined estrogen and progestogen preparations, which could substantially increase the breast cancer risk compared with non-users. Third, high mammographic density has been associated with an increased risk of breast cancer due to the abundant epithelial tissue where cancer could arise and to the masking bias that reduces the sensitivity of mammography. In addition, increasing age, personal history of breast pathologies (such as atypical hyperplasia and lobular carcinoma in situ), exposure to therapeutic chest radiation, reproductive factors (early menarche, low parity, shorter breastfeeding periods, and late menopause), high body mass index, alcohol, and inadequate physical activity are other clinical risk factors for breast tumor [38,39], which could be correlated with radiomic data in future studies. In our study, the predictive model that was obtained with the RF classifier (AUC = 0.59, 95% CI 0.57–0.60) and with SVM-RBF (AUC = 0.56, 95% CI 0.55–0.57) showed only limited potential in discriminating between benign and malignant microcalcifications. However, we are confident that the integration with other parameters, such as clinical factor risks, may improve the performance of a radiomic classification model in the recognition of microcalcifications that need a VABB.

Our study has some limitations. First, it is a retrospective single-center study; therefore, a multicentric study would be important to assess if these results are valid on a larger scale and could be generalized. Second, the data set should be increased, although we did not have a small sample size compared with similar studies found in the literature. Considering the relatively small sample, the training and validation were performed using the 12 representative features out of the 91 extracted radiomic features to avoid overfitting. A larger data set would allow us to assess the performance of radiomic classification models featuring a higher number of radiomic features without over-fitting issues. Third, the process of manual segmentation involved a single radiologist expert in breast radiology. This brings to attention the impossibility of evaluating the reliability of the intra- and inter-observer processes. Multiple radiologists would provide a closer approximation of the average segmentation, which would, in turn, reduce the variability in the model. Fourth, the clinical risk factors were not included. Future studies are needed to assess if the integration of data, such as genetic factors and the use of menopausal hormone therapies, improves the radiomic model. Lastly, the radiomic results were not correlated with breast density, which can influence the risk of developing breast cancer and, therefore, modify the distinction between benign and malignant calcifications; however, studies have shown that radiomics reflect the intrinsic properties of mammographic parenchymal complexity beyond conventional breast density measures and may provide additional information for risk assessment [39,40].

## 5. Conclusions

According to the present study results, radiomic features alone are not able to define the clinical management of patients with BI-RADS category 4 microcalcifications. However, our results did not exclude that a further improved classification model can reduce the false-positive rate and adjust the radiologic cut-off for image-guided breast biopsy. We believe that with further large-scale studies capable of overcoming the limits of our work, it might be possible to obtain a radiomic classification model as a supplement to the BI-RADS for a better selection of patients with suspicious microcalcifications that need a VABB.

## Figures and Tables

**Table 1:** The table shows how the patients were divided into two groups: one addressed to follow up and the other addressed to surgical excision, based on the histopathological category of the biopsy sample; DBT-VABB: digital breast tomosynthesis-guided vacuum-assisted breast biopsy.

DBT-VABB Histopathologic Reports			
B2	B3	B5a	B5b
126	45	63	18
Total 126	Total 126		
Follow up	Surgical Excision		

**Table 2:** Performance measures of the four ML radiomic classifiers for benign calcification versus malignant calcification.

Classification Methods	Sensitivity	Specificity	Accuracy	AUC
Linear support vector classifier	0.53 [0.50–0.56]	0.45 [0.42–0.48]	0.48 [0.46–0.50]	0.49 [0.48–0.50]
Radial basis function support vector classifier	0.57 [0.55–0.59]	0.55 [0.53–0.57]	0.56 [0.54–0.57]	0.56 [0.55–0.57]
Logistic regression	0.45 [0.41–0.49]	0.50 [0.46–0.55]	0.46 [0.45–0.48]	0.48 [0.46–0.49]
Random forest	0.56 [0.54–0.58]	0.61 [0.59–0.63]	0.58 [0.57–0.59]	0.59 [0.57–0.60]

## Data Availability

The data presented in this study are available on request from the corresponding author. The data are not publicly available due to ethical reasons.

## Abbreviations

AUC	area under the curve
BI-RADS	Breast Imaging Reporting and Data System
CI	confidence interval
CV	cross-validation
DBT	digital breast tomosynthesis
DCE-MRI	dynamic contrast-enhanced magnetic resonance imaging
DCIS	ductal carcinoma in situ
GLCM	gray level co-occurrence matrix
GLDM	gray level dependence matrix
GLRLM	gray level run length matrix
GLSZM	gray level size zone features
IBSI	Image Biomarker Standardization Initiative
LR	logistic regression
ML	machine-learning
NCB	needle core biopsy
NGTDM	neighboring gray tone difference matrix
RBF	radial basis function
RF	random forest
ROI	region of interest
SVM	support vector machine
VABB	vacuum-assisted breast biopsy
